# Hemorrhagic Anuria With Acute Kidney Injury After a Single Dose of Acetazolamide: A Case Study of a Rare Side Effect

**DOI:** 10.7759/cureus.10107

**Published:** 2020-08-29

**Authors:** Christy Lawson, Leisa Morris, Vera Wilson, Bracken Burns

**Affiliations:** 1 Surgery, Quillen College of Medicine, East Tennesse State University, Johnson City, USA; 2 Trauma, Ballad Health Trauma Services, Johnson City, USA; 3 Pharmacy, Ballad Health Trauma Services, Johnson City, USA; 4 Surgery, Quillen College of Medicine, East Tennessee State University, Johnson City, USA

**Keywords:** acetazolamide, hemorrhagic anuria, acute kidney injury

## Abstract

Acetazolamide (ACZ) is a relatively commonly used medication in critical illness, glaucoma and altitude sickness. ACZ is sometimes used in the intensive care unit to assist with the treatment of metabolic alkalosis in ventilated patients. This is a case report of a patient who received two doses of ACZ, one week apart, for metabolic alkalosis and subsequently developed renal colic and dysuria that progressed to hemorrhagic anuria and acute kidney injury. This is an incredibly rare side effect of ACZ therapy, and has been reported in a few case reports in the literature, but usually is associated with a longer duration of therapy. This case resolved entirely within 24 hours with aggressive fluid therapy. Clinicians using ACZ therapy for any reason should be aware of this rare but significant side effect.

## Introduction

Acetazolamide (ACZ) is a carbonic anhydrase inhibitor. It works to cause an accumulation of carbonic acid in the proximal kidney, preventing its breakdown, and causes lowering of blood pH and resorption of sodium, bicarbonate, and chloride with their subsequent excretion into the urine [1]. It is given either by oral or intravenous means, in either 125mg, 250mg or 500mg doses [2]. It is used in the treatment of glaucoma, altitude sickness, periodic paralysis, epilepsy, congestive heart failure, and idiopathic intracranial hypertension [1]. Additionally, it is often utilized in an off-label manner in patients on mechanical ventilation who have primary or mixed metabolic alkalosis as an aid to help correct pH, and allow the patient more efficient ventilator weaning [3].

ACZ has a number of very common side effects, such as nausea, vomiting, fatigue, abdominal pain, and paresthesias. Rarer, more severe side effects have been described, such as Stevens-Johnson Syndrome [2]. There have been a few case reports in the literature, dating back to the 1950s, that describe hemorrhagic anuria and acute kidney injury (AKI) as a rare side effect of ACZ, but this has typically been seen in patients who receive over 1200mg within 48 hours [4].

## Case presentation

A 38-year-old male with no significant past medical history who took no medications and had allergies to penicillin, cefepime, and cantaloupe, fell backward from the bed of a moving truck onto his head. He sustained a significant blunt head trauma, with bilateral subdural hematomas, temporal bone fracture, nasal and orbital bone fractures, effacement of cisterns, cerebral compression, and possible diffuse axonal injury. He was intubated and placed on mechanical ventilation for airway protection due to his severe traumatic brain injury and coma, and placed in the surgical intensive care unit. His hospital course was relatively uncomplicated, though he did remain on the ventilator for 12 days and required tracheostomy placement due to prolonged decreased mental status. After tracheostomy placement on hospital day 8, he was being weaned fairly aggressively from the ventilator and was showing signs of improvement from a mental status perspective.

On hospital day 9, the patient had an arterial blood gas that revealed a pH of 7.53, a pCO2 of 38, a pO2 of 67, a bicarbonate level of 28 with a base excess of +4. In an effort to help his primarily metabolic alkalosis, a single dose of ACZ was given, 500mg intravenously. He had received the same 500mg intravenous dose of ACZ on hospital day 3 for a similar indication, a week prior to this dose. At this point, he had an indwelling foley catheter, a normal creatinine, and was producing over 2 L a day of clear urine.

On hospital day 11, the patient was successfully on tracheostomy collar and was more lucid and able to communicate relatively clearly. He began complaining of severe bilateral flank pain with dysuria and had a significantly decreased urine output of only 275mL of cloudy, sediment containing urine. A urinalysis revealed red cells, no white cells, no nitrates, and no bacteria. He had some mucoid-like discharge from the indwelling foley catheter with irrigation. A computed tomography of the abdomen and pelvis with renal stone protocol was obtained to evaluate for kidney stones. The images revealed no evidence of obstructive uropathy, a decompressed bladder, and several small, nonobstructing renal calculi in bilateral kidney lower poles. The urinary catheter was exchanged and the bladder was irrigated. Only the irrigation and some blood-stained mucous was evacuated. Urinary analgesia was given to the patient and aggressive fluid therapy was started. His creatinine, which had been 0.48 on the morning laboratory panel, increased to 2.0 on an evening laboratory panel with a blood urea nitrogen (BUN):creatinine ratio of 19.5:1, which can be interpreted as normal or can also indicate post-renal disease.

Due to the fact that the patient remained afebrile, had been on aztreonam for suspected pneumonia, and had no findings on urinalysis to indicate a urinary tract infection, a thorough medication review for nephrotoxic medications was conducted. The patient had received a 24-hour course of vancomycin on hospital day 4-5, as part of empiric therapy for a fever and leukocytosis workup. It had been stopped on hospital day 5, with appropriate monitoring and clearing of troughs. No other nephrotoxic medications had been administered during the patient’s hospital course.

**Figure 1:**
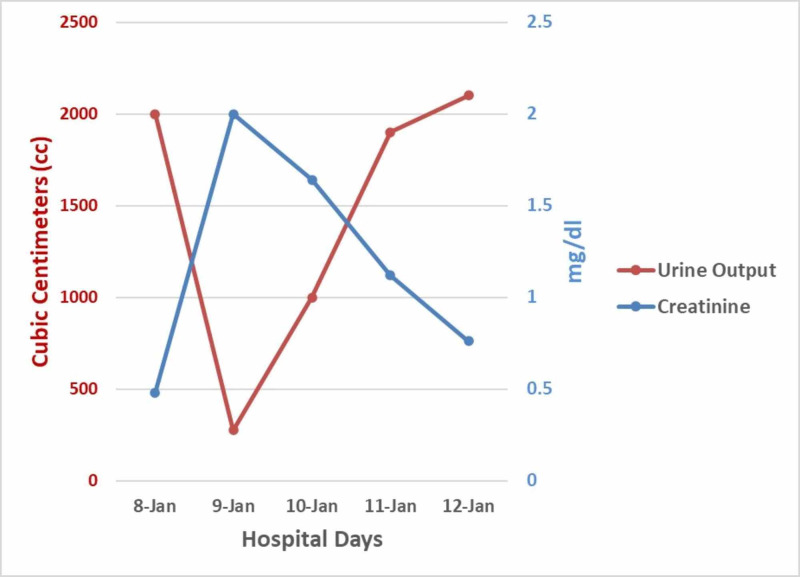
Correlation Between Creatinine and Urine Output from Hospital Day 11 to Hospital Day 15

A literature search revealed the case reports on hemorrhagic anuria and AKI with ACZ administration, as documented in the discussion, so fluid therapy was initiated. The following day, hospital day 10, the patient had near complete resolution of his renal colic and dysuria symptoms, had returned to a normal urine output of 1000mL in 12 hours and was producing clear urine again. His creatinine had decreased to 1.64. Over the next 48 hours, his creatinine returned to normal and his urine output also returned to normal. See Figure 1 for a representation of urine output and creatinine over the duration of his brief course of AKI.

## Discussion

This rare side effect of ACZ has been discussed in several case reports or case series throughout the decades. The first mention of renal lesions from ACZ administration was documented by Glushien and Fisher in 1956. In their paper, a patient with Hodgkin’s disease received a four-day course of ACZ at 750mg per day, for a total of 3g of the medication. In this patient, the ACZ was utilized as a diuretic. Postmortem, the patient was found to have coagulated blood products and sulfonamide crystals similar in appearance to crystallized ACZ within the renal tubules [5].

The next mention of a patient with renal impairment after ACZ administration also mentions this sulfonamide crystalluria. This was found in a 59-year-old patient who received a six-day course of ACZ (unknown dosing) for glaucoma treatment. The patient reported severe renal colic type pain, hematuria and had azotemia and anuria on clinical assessment, without evidence of kidney stones. He also had bilateral ureteral obstruction requiring catheterization. This patient responded to oral bicarbonate therapy, fluid resuscitation, and urinary catheterization, with return to baseline renal function by discharge [6].

In a 1975 case study, Howlett described a 69-year-old patient who received a two-week course of ACZ, 1000mg per day, for glaucoma. He presented several days later with anuria, azotemia, nausea, and severe flank pain. Urinalysis revealed red blood cells, but this case differed in the fact that they did not discover sulfonamide crystals within the urine. Again, the patient responded to oral bicarbonate therapy and fluid resuscitation, with normalization of kidney function [7]. Several other patients were described in a case series in 1978, with clinical presentations and laboratory assessments similar to the aforementioned patients who also received high dose ACZ for glaucoma management. They again had restoration of kidney function with oral bicarbonate and aggressive hydration. In this case series, the patients had antegrade urograms that revealed dilated renal pelvises with mucosal swelling and sludge-like material within the ureters that were causing blockages [8].

In a 1989 paper on ACZ-induced AKI, a patient who presented after taking high-dose ACZ, again for glaucoma, presented with the previously described symptoms of severe flank pain, azotemia, nausea, and vomiting with AKI. This patient had no radiographic evidence of obstruction of the renal system, and underwent a kidney biopsy. This biopsy demonstrated tubular damage with cellular debris and intratubular crystal deposition. This patient also responded well to supportive measures with fluid rehydration, and this kidney biopsy that demonstrated crystal deposits similar to that found on the previous study patients’ assessments lends further proof to the idea that ACZ can cause renal impairment, theoretically due to this crystal deposition within the renal tubules [9].

In all the case studies described, the patients were receiving high-dose ACZ therapy. The only case study that demonstrated similar findings with low-dose ACZ therapy is a 2014 case study that describes a patient who developed severe AKI and bilateral flank pain after ACZ administration for high altitude sickness. He received a total dose of 1200mg in 48 hours of ACZ, a far lower dose than previously described. This patient also had no radiographic evidence of ureteral obstruction. All other tests done on this patient to elucidate the cause of the renal failure were negative. The patient required two sessions of hemodialysis and aggressive fluid resuscitation, but subsequently had return to normal renal function [10].

The patient described in this case study received two single 500mg doses of ACZ, a week apart, yet presented almost identically to these previously described cases. He had flank pain, azotemia, AKI, and red blood cells on urinalysis. He also had no evidence of ureteral obstruction on CT imaging. On urinary catheterization and irrigation of the bladder, he was noted to have mucoid discharge and significant sediment within the irrigation fluid, without urine output. This fluid was, unfortunately, not sent for analysis, so the nature of the sediment is not described. The only other nephrotoxic medication this patient had received was a 24-hour course of vancomycin, however this had been discontinued full six days prior to the presentation of AKI. Perhaps the most clear similarity in this patient’s presentation to those cases of AKI attributed to ACZ described in the literature was his complete and rapid resolution of symptoms and the return of his creatinine to baseline with IV fluid hydration.

## Conclusions

The “classic” presentation of ACZ-induced “hemorrhagic anuria” includes anuria, bilateral, severe flank pain, red cells on urinalysis, and no radiographic evidence of ureteral obstruction. This has been described rather robustly in the literature in the form of case series or studies, and is generally reported with high-dose ACZ administration. Our case illustrates that this presentation can occur with even a single, relatively low dose of ACZ, even in a patient with no pre-existing renal dysfunction. Clinicians should be aware of this rare side effect with the potential for significant morbidity, as well as the concept that supportive measures with aggressive fluid rehydration and possibly oral bicarbonate can rapidly reverse the AKI seen in this patient population.
